# Supplementary material for: Effects of Cannabidiol Supplementation on Skeletal Muscle Regeneration after Intensive Resistance Training
Source: Nutrients. 2021 Aug 30;13(9):3028. doi: 10.3390/nu13093028 (PMC8469280; doi:10.3390/nu13093028)
Supplement: Supplementary file 1 [file nutrients-13-03028-s001.zip › nutrients-1347771-supplementary.pdf]

Table S1. Effects of recovery interval (RI T 0—RI T 72), of Δt, and of CBD on ln (CK) [U/l]. RI T 0 × CBD through RI T 72 × CBD indicate interaction effects. SE: standard error; DF: degrees of freedom; t: t-value; *p*: *p*-value (*= $p<0.05$, **= $p<0.01$, ***= $p<0.001$, n.s.: not significant); ES: effect size.

	Value	SE	DF	t	*p*	ES
(Intercept)	5.43	0.175	352	31	***	-
RI T 24	0.794	0.123	352	6.44	***	0.687
RI T 48	0.637	0.128	352	4.98	***	0.531
RI T 72	0.683	0.141	352	4.85	***	0.517
Δ t	−0.00018	6.69 e^{-5}	352	−2.69	**	−0.286
RI T 0 × CBD	−0.0222	0.129	352	−0.172	n.s.	-
RI T 24 × CBD	−0.17	0.129	352	−1.32	n.s.	-
RI T 48 × CBD	−0.204	0.129	352	−1.59	n.s.	-
RI T 72 × CBD	−0.287	0.13	352	−2.21	*	−0.236

Table S2. Effects of recovery interval (RI T 0—RI T 72), of Δ t, and of CBD on ln (Myo) [ng / ml]. RI T 0 × CBD through RI T 72 × CBD indicate interaction effects. SE: standard error; DF: degrees of freedom; t: t-value; *p*: *p*-value (*= $p<0.05$, **= $p<0.01$, ***= $p<0.001$, n.s.: not significant); ES: effect size.

	Value	SE	DF	t	*p*	ES
(Intercept)	3.56	0.121	352	29.5	***	-
RI T 24	0.289	0.103	352	2.82	**	0.301
RI T 48	0.281	0.106	352	2.64	**	0.281
RI T 72	0.459	0.118	352	3.91	***	0.417
Δ t	−0.000118	5.01 e^{-5}	352	−2.35	*	−0.25
RI T 0 × CBD	−0.0656	0.111	352	−0.59	n.s.	-
RI T 24 × CBD	−0.176	0.111	352	−1.59	n.s.	-
RI T 48 × CBD	−0.174	0.111	352	−1.57	n.s.	-
RI T 72 × CBD	−0.225	0.112	352	−2.01	*	−0.214

Table S3. Effects of recovery interval (RI T 0—RI T 72), of Δ t, and of CBD on 1RM [kg]. RI T 0 × CBD through RI T 72 × CBD indicate interaction effects. SE: standard error; DF: degrees of freedom; t: t-value; *p*: *p*-value (*= $p<0.05$, **= $p<0.01$, ***= $p<0.001$, n.s.: not significant); ES: effect size.

	Value	SE	DF	t	*p*	ES
(Intercept)	116	6.03	166	19.3	***	-
RI T 24	−3.84	1.23	166	−3.12	**	−0.484
RI T 48	−1.39	1.22	166	−1.13	n.s.	-
RI T 72	−2.34	1.27	166	−1.84	n.s.	-
Δ t	0.00163	0.00074	166	2.2	*	0.342
RI T 0 × CBD	0.318	0.913	166	0.348	n.s.	-
RI T 24 × CBD	2.9	1.57	166	1.85	n.s.	-
RI T 48 × CBD	1.17	1.55	166	0.753	n.s.	-
RI T 72 × CBD	3.75	1.57	166	2.39	*	0.371

Table S4. Effects of recovery interval (RI T 0—RI T 72), of Δ t, and of CBD on CMJ [cm]. RI T 0 × CBD through RI T 72 × CBD indicate interaction effects. SE: standard error; DF: degrees of freedom; t: t-value; *p*: *p*-value (***= $p<0.001$, n.s.: not significant); ES: effect size.

	Value	SE	DF	t	*p*	ES
(Intercept)	55.8	1.68	167	33.1	***	-
RI T 24	−0.829	0.533	167	−1.55	n.s.	-
RI T 48	−0.125	0.53	167	−0.236	n.s.	-

RI T 72	−0.608	0.551	167	−1.1	n.s.	-
RI T 0 × CBD	0.0585	0.396	167	0.148	n.s.	-
RI T 24 × CBD	−0.172	0.679	167	−0.254	n.s.	-
RI T 48 × CBD	−0.743	0.674	167	−1.1	n.s.	-
RI T 72 × CBD	1.17	0.68	167	1.72	n.s.	-

Table S5. Individual CK values for all time points and each group [in U / L]. Δ values are colored according to their values: Increase; Decrease; No Change. PLA = Placebo; CBD = Cannabidiol; CK = Skeletal Muscle Specific Creatine Kinase; Δ = POST −PRE.

	PLA									CBD								
	T24			T48			T72			T24			T48			T72		
Subjects	PRE	POST	Δ	PRE	POST	Δ	PRE	POST	Δ	PRE	POST	Δ	PRE	POST	Δ	PRE	POST	Δ
1	154	202	48	154	267	113	2336	471		198	201	3	208	181	−27	516	158	−358
2	306	540	234	107	192	85	904	23,801		348	295	−53	379	515	136	199	246	47
3	196	539	343	162	284	122	152	227		187	773	586	279	242	−37	157	172	15
4	147	598	451	60	180	120	93	530		347	277	−70	117	426	309	83	430	347
5	242	322	80	217	161	−56	136	475		77	321	244	104	243	139	143	130	−13
6	167	245	78	134	452	318	111	195		167	213	46	125	194	69	119	215	96
7	138	177	39	159	134	−25	99	107		125	186	61	99	151	52	104	120	16
8	221	333	112	163	178	15	248	166		165	166	1	110	146	36	284	143	−141
9	119	156	37	322	250	−72	126	157		200	185	−15	95	160	65	99	127	28
10	230	400	170	195	252	57	244	221		234	297	63	201	236	35	209	201	−8
11	145	227	82	183	1018	835				207	410	203	343	368	25	133	153	20
12	368	1211	843	738	480	−258	141	308	167	132	361	229	361	215	−146	232	230	−2
13	235	713	478	174	437	263	116	617	501	240	303	63	167	1201	1034	243	592	349
14	235	201	−34	93	117	24	109	140	31	96	221	125	103	151	48	119	126	7
15	281	267	−14	97	98	1	127	23,471	23,344	246	1097	851	112	225	113	135	467	332
16	201	267	66	191	3992	3801	277	379	102	190	318	128	214	481	267	135	211	76

Table S6. A. Individual Myo values for all time points and each group [in ng / mL]. Δ values are colored according to their values: Increase; Decrease; No Change. PLA = Placebo; CBD = Cannabidiol; Myo = Myoglobin; Δ = POST −PRE.

	PLA									CBD								
	T24			T48			T72			T24			T48			T72		
Subjects	PRE	POST	Δ	PRE	POST	Δ	PRE	POST	Δ	PRE	POST	Δ	PRE	POST	Δ	PRE	POST	Δ
1	25	25	0	25	25	0	80	25		25	25	0	25	25	0	25	26	1
2	29	42	13	25	25	0	204	319		25	25	0	25	25	0	25	26	1
3	25	31	6	25	33	8	25	27		25	41	16	29	32	3	27	27	0
4	42	65	23	27	42	15	36	77		58	33	−25	31	118	87	29	55	26
5	26	28	2	25	25	0	25	42		25	28	3	25	30	5	25	25	0
6	28	31	3	27	25	−2	36	35		31	25	−6	25	30	5	25	26	1
7	30	25	−5	25	25	0	25	25		25	28	3	25	26	1	25	42	17
8	33	43	10	36	34	−2	35	29		26	25	−1	25	32	7	26	34	8
9	25	25	0	52	31	−21	25	32		28	28	0	26	29	3	27	25	−2
10	25	34	9	25	33	8	35	25		25	25	0	25	31	6	29	31	2
11	25	41	16	25	85	60				26	47	21	29	34	5	25	25	0
12	50	59	9	55	28	−27	25	34	9	27	30	3	49	25	−24	29	31	2
13	46	71	25	35	44	9	31	59	28	32	42	10	35	76	41	45	57	12

14	27	25	−2	25	25	0	25	25	0	25	25	0	25	25	0	25	25	0
15	32	25	−7	25	29	4	25	1920	1895	79	49	−30	26	25	−1	25	51	26
16	41	45	4	27	579	552	40	35	−5	28	33	5	25	29	4	25	35	10

Table S7. Individual 1RM Back Squat values for all time points and each group [in kg]. Δ values are colored according to their values: Increase; Decrease; No Change. PLA = Placebo; CBD = Cannabidiol; 1RM = One-Repetition Maximum; Δ = POST −PRE.

	PLA									CBD								
	T24			T48			T72			T24			T48			T72		
**Subjects**	**PRE**	**POST**	**Δ**	**PRE**	**POST**	**Δ**	**PRE**	**POST**	**Δ**	**PRE**	**POST**	**Δ**	**PRE**	**POST**	**Δ**	**PRE**	**POST**	**Δ**
1	87.5	85	−2.5	80	82.5	2.5	85	82.5		85	87.5	2.5	85	85	0	80	82.5	2.5
2	100	92.5	−7.5	100	102.5	2.5	102.5	102.5		102.5	107.5	5	107.5	107.5	0	100	100	0
3	95	90	−5	90	95	5	85	82.5		87.5	85	−2.5	90	92.5	2.5	92.5	95	2.5
4	127.5	120	−7.5	122.5	122.5	0	125	125		125	117.5	−7.5	127.5	120	−7.5	125	120	−5
5	145	130	−15	150	155	5	155	137.5		147.5	145	−2.5	140	145	5	145	145	0
6	147.5	140	−7.5	140	140	0	135	130		135	135	0	145	135	−10	140	140	0
7	112.5	110	−2.5	110	105	−5	110	105		115	110	−5	112.5	112.5	0	110	115	5
8	165	150	−15	160	165	5	160	170		155	155	0	160	160	0	165	160	−5
9	130	132.5	2.5	140	137.5	−2.5	145	150		140	140	0	132.5	127.5	−5	130	130	0
10	150	150	0	160	152.5	−7.5	147.5	147.5		150	150	0	152.5	152.5	0	150	160	10
11	100	100	0	105	105	0				105	105	0	105	100	−5	100	105	5
12	87.5	85	−2.5	92.5	70	−22.5	87.5	90	2.5	95	90	−5	90	95	5	100	100	0
13	110	115	5	115	110	−5	110	110	0	115	120	5	115	115	0	120	120	0
14	105	105	0	100	105	5	105	100	−5	107.5	107.5	0	110	110	0	107.5	110	2.5
15	100	100	0	107.5	107.5	0	105	100	−5	105	105	0	107.5	105	−2.5	110	112.5	2.5
16	125	130	5	120	105	−15	115	110	−5	120	120	0	125	125	0	125	125	0

Table S8. A. Individual CMJ values for all time points and each group [in cm]. Δ values are colored according to their values: Increase; Decrease; No Change. PLA = Placebo; CBD = Cannabidiol; CMJ = Countermovement Jump; Δ = POST −PRE.

	PLA									CBD								
	T24			T48			T72			T24			T48			T72		
**Subjects**	**PRE**	**POST**	**Δ**	**PRE**	**POST**	**Δ**	**PRE**	**POST**	**Δ**	**PRE**	**POST**	**Δ**	**PRE**	**POST**	**Δ**	**PRE**	**POST**	**Δ**
1	45.3	44.6	−0.7	39.0	40.0	1.0	46.0	44.0		43.6	44.3	0.7	43.3	45.0	1.7	43.0	47.6	4.6
2	54.0	55.3	1.3	55.0	57.3	2.3	56.3	59.0		59.0	59.3	0.3	57.6	56.0	−1.6	56.6	55.0	−1.6
3	57.0	56.3	−0.7	55.6	59.6	4.0	55.0	51.0		56.0	55.0	−1.0	55.3	53.6	−1.7	55.3	57.3	2.0
4	63.3	63.0	−0.3	66.3	66.3	0.0	65.0	63.3		62.0	62.3	0.3	62.6	55.3	−7.3	63.3	61.6	−1.7
5	65.3	62.0	−3.3	65.0	67.6	2.6	65.0	64.6		65.0	61.6	−3.4	64.3	64.6	0.3	62.6	65.3	2.7
6	62.6	59.0	−3.6	58.3	58.6	0.3	57.0	60.3		60.6	58.6	−2.0	60.6	58.6	−2.0	60.6	59.3	−1.3
7	54.3	53.6	−0.7	52.0	53.0	1.0	53.0	52.3		52.0	51.3	−0.7	52.6	54.0	1.4	55.0	53.0	−2.0
8	59.0	60.0	1.0	65.3	63.0	−2.3	61.3	65.0		59.3	61.6	2.3	62.0	63.6	1.6	65.0	63.0	−2.0
9	46.0	47.6	1.6	46.0	46.0	0.0	43.6	48.3		47.6	47.0	−0.6	44.5	45.0	0.5	50.0	49.0	−1.0
10	69.6	62.0	−7.6	64.3	65.3	1.0	65.6	64.6		64.3	62.6	−1.7	65.6	66.6	1.0	64.3	67.6	3.3
11	60.6	56.0	−4.6	56.0	57.3	1.3				51.3	49.0	−2.3	54.0	52.0	−2.0	55.6	56.6	1.0
12	52.0	49.6	−2.4	53.0	47.0	−6.0	50.6	46.3	−4.3	52.0	49.0	−3.0	49.3	49.3	0.0	49.6	53.3	3.7
13	61.6	59.3	−2.3	57.3	56.3	−1.0	58.0	52.3	−5.7	57.3	56.6	−0.7	61.0	58.5	−2.5	59.6	58.6	−1.0
14	49.6	50.0	0.4	51.6	50.3	−1.3	46.0	48.0	2.0	48.0	49.0	1.0	49.0	48.0	−1.0	46.6	47.6	1.0

15	47.3	48.6	1.3	48.0	49.0	1.0	50.6	49.0	−1.6	50.3	49.3	−1.0	50.6	49.3	−1.3	51.0	47.0	−4.0
16	44.0	46.0	2.0	46.0	42.6	−3.4	43.6	43.0	−0.6	42.3	41.3	−1.0	45.6	45.0	−0.6	46.6	46.3	−0.3

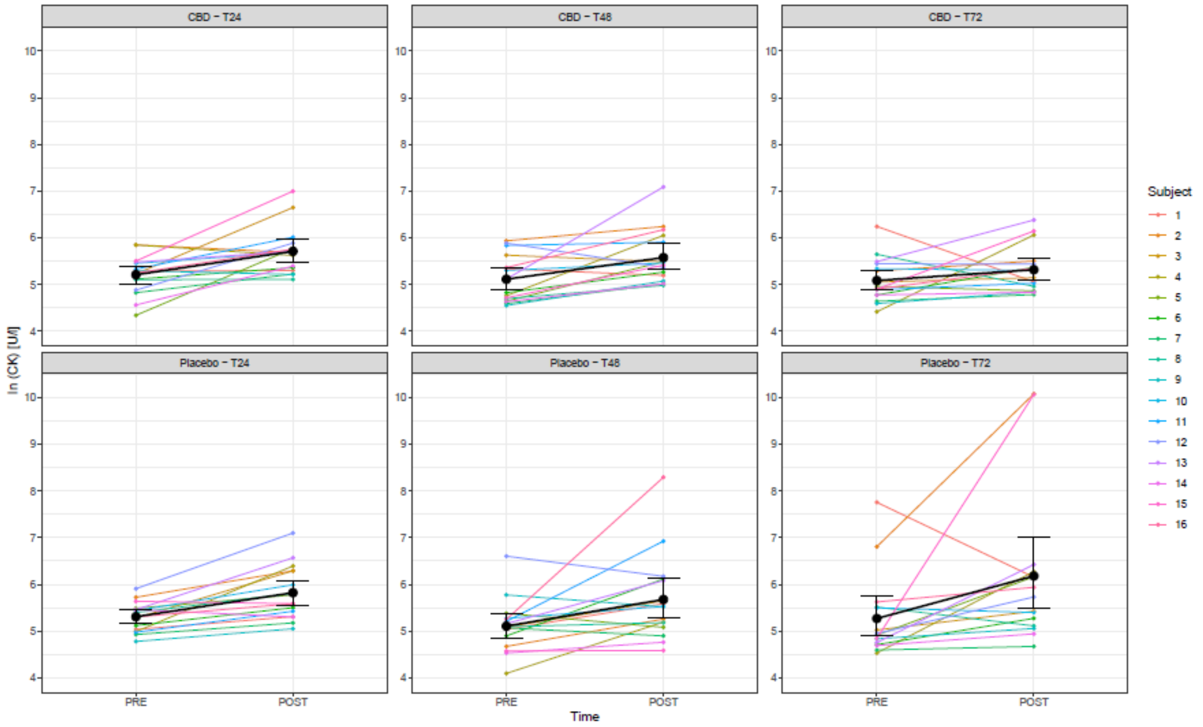

Figure S1. Individual ln (CK) trends (coloured) and the mean ±95% confidence interval (black) for each group and time point (indicated above each panel). CK = Skeletal Muscle Specific Creatine Kinase; ln = Logarithm.

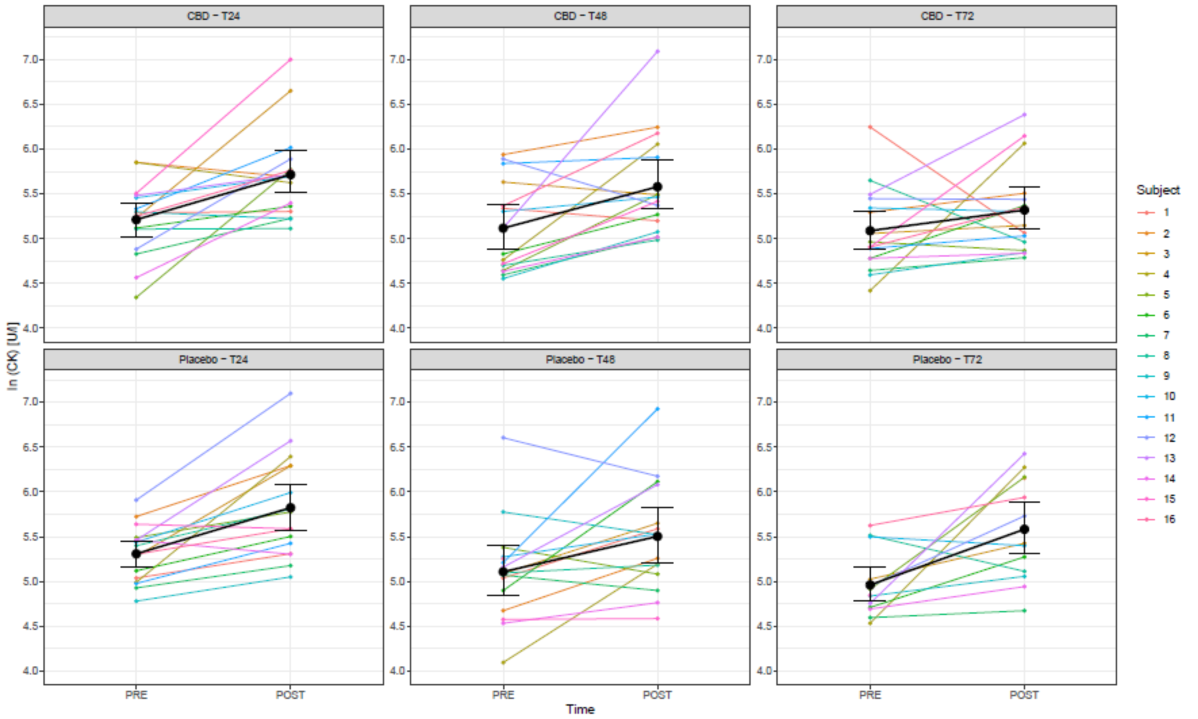

Figure S2. Individual ln (CK) trends (colored) and the mean ±95% confidence interval (black) for each group and time point (indicated above each panel) cut at ln (CK) = 7.5 to eliminate outliers for better visibility of changes over time. Excluded values: Placebo—T48 = Subject 16; Placebo—T72: Subjects 1, 2, and 15. CK = Skeletal Muscle Specific Creatine Kinase; ln = Logarithm.

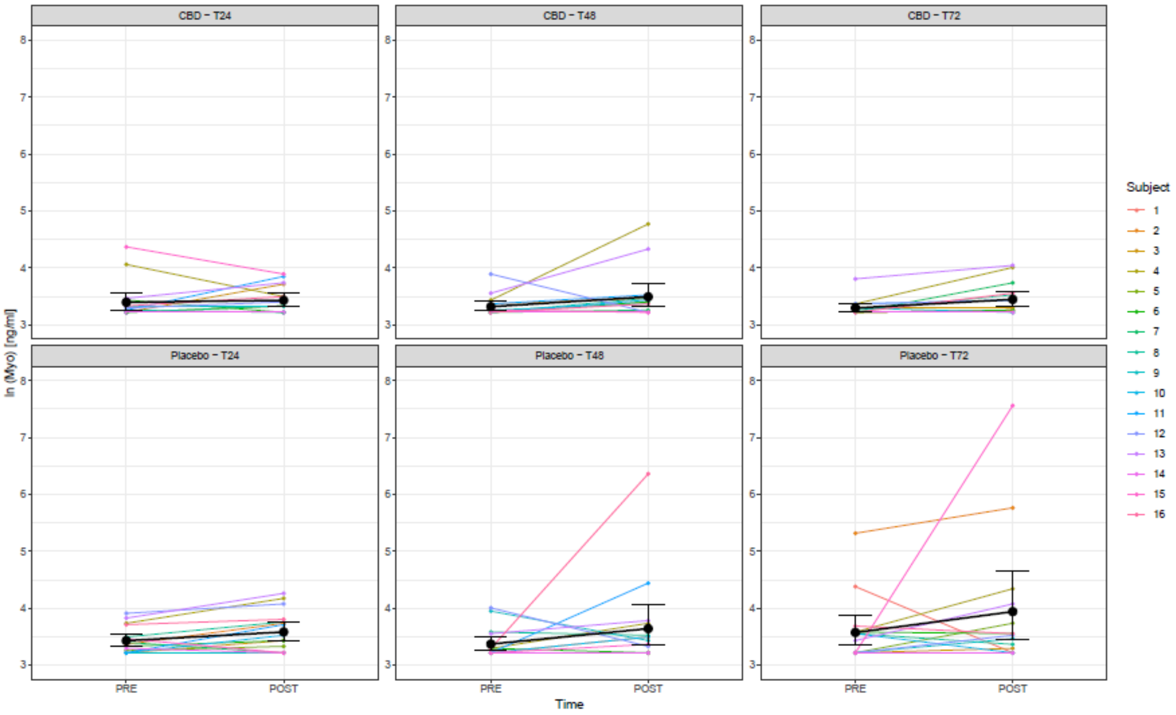

Figure S3. Individual ln (Myo) trends (colored) and the mean ±95% confidence interval (black) for each group and time point (indicated above each panel). Myo = Myoglobin; ln = Logarithm.

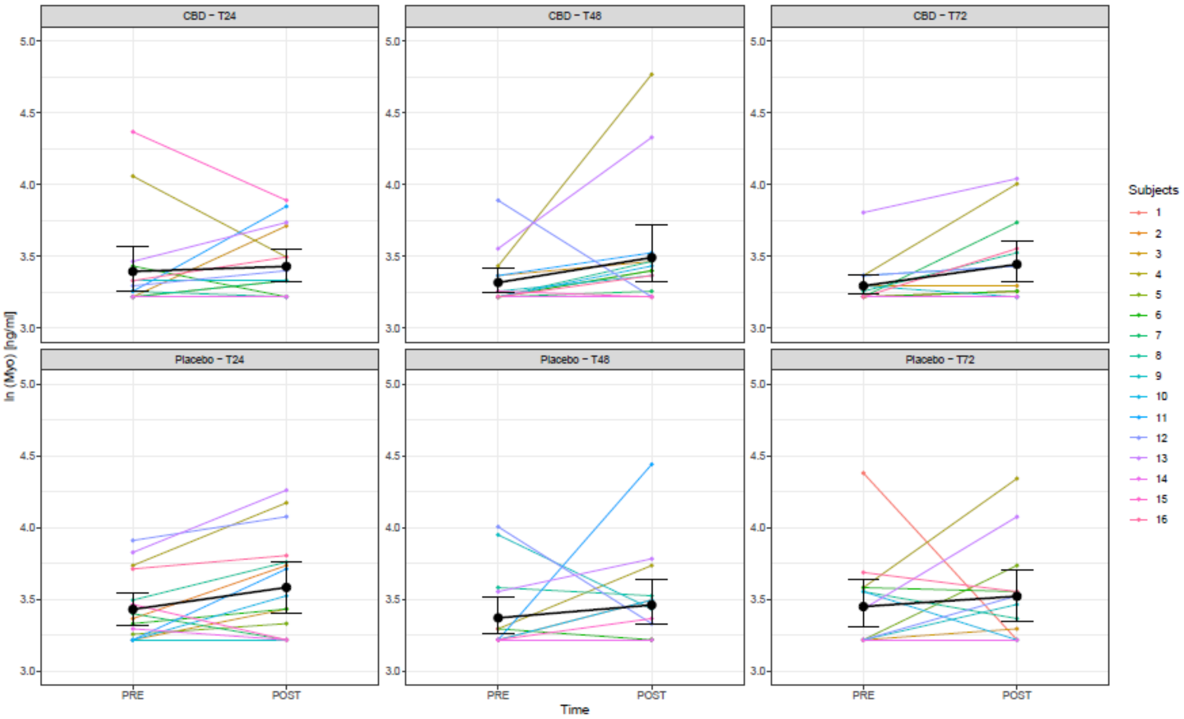

Figure S4. Individual ln (Myo) trends (coloured) and the mean ± 95% confidence interval (black) for each group and time point (indicated above each panel) cut at ln (Myo) = 5.0 to eliminate outliers for better visibility of changes over time. Excluded values: Placebo—T48 = Subject 16; Placebo—T72: Subjects 2 and 15. Myo = Myoglobin; ln = Logarithm.

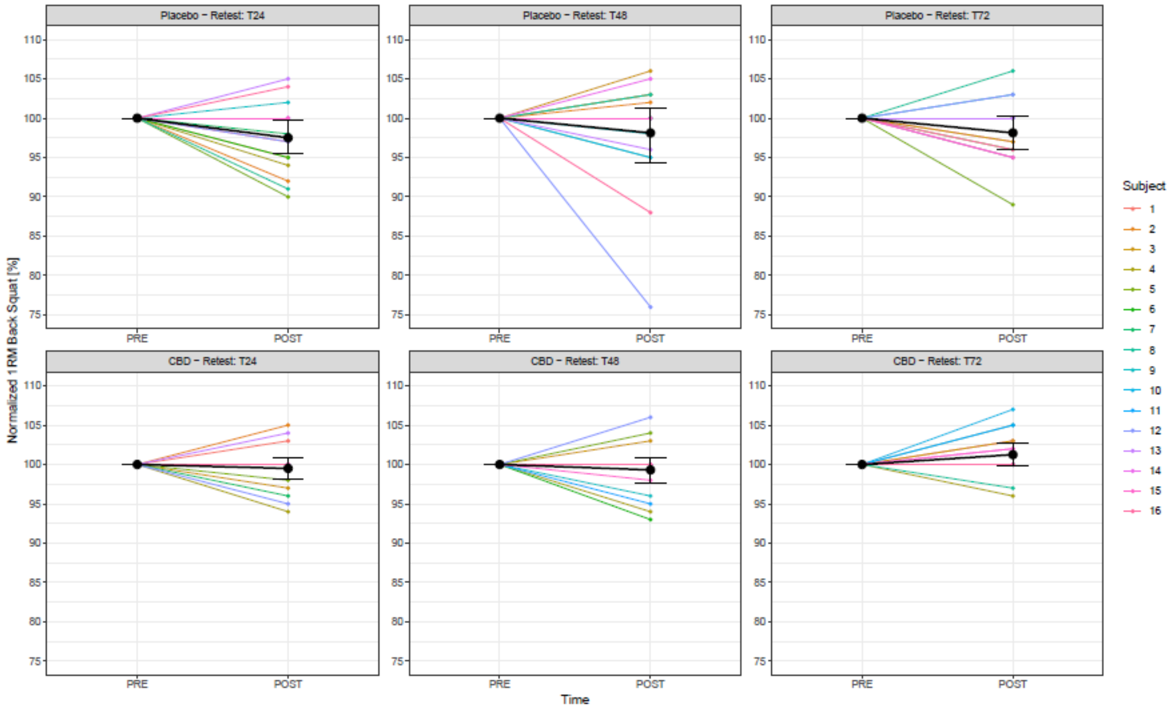

Figure S5. Individual 1RM Back Squat trends (coloured) and the mean ±95% confidence interval (black) for each group and time point (indicated above each panel). 1RM = One-Repetition Maximum.

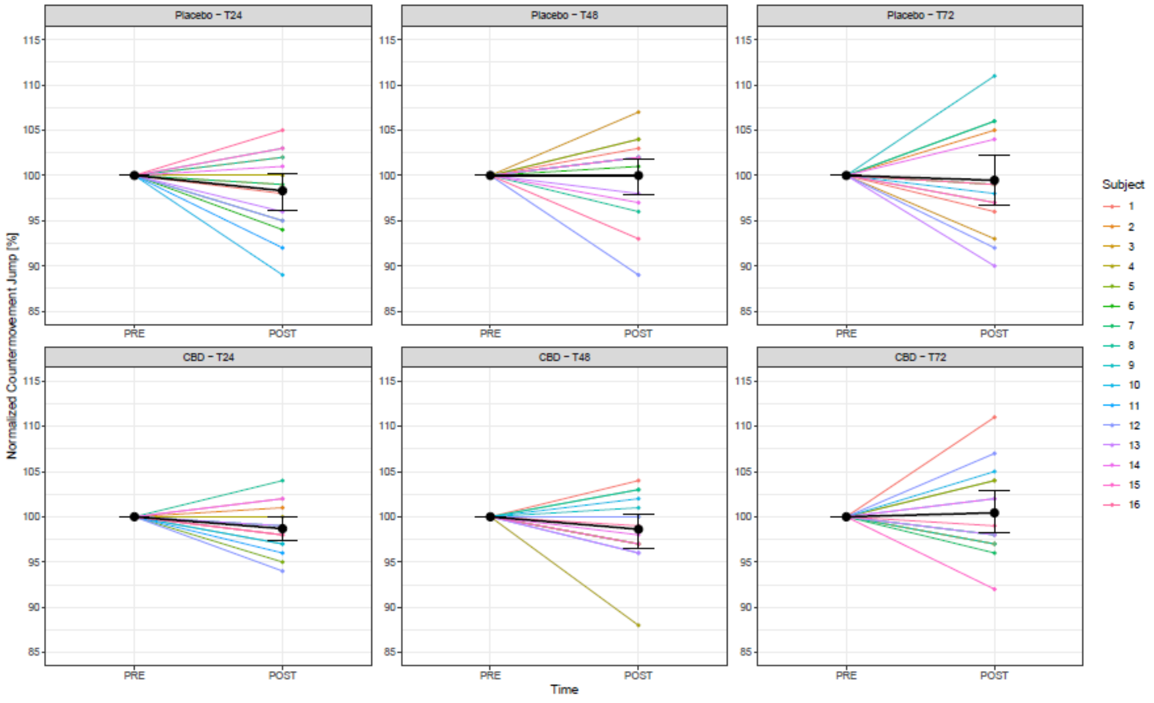

Figure S6. Individual CMJ trends (coloured) and the mean ±95% confidence interval (black) for each group and time point (indicated above each panel). CMJ = Countermovement Jump.
